# CRISPR/Cas12a-Enhanced Loop-Mediated Isothermal Amplification for the Visual Detection of *Shigella flexneri*

**DOI:** 10.3389/fbioe.2022.845688

**Published:** 2022-02-21

**Authors:** Yaoqiang Shi, Lan Kang, Rongrong Mu, Min Xu, Xiaoqiong Duan, Yujia Li, Chunhui Yang, Jia-Wei Ding, Qinghua Wang, Shilin Li

**Affiliations:** ^1^ Provincial Key Laboratory for Transfusion-Transmitted Infectious Diseases, Institute of Blood Transfusion, Chinese Academy of Medical Sciences and Peking Union Medical College, Chengdu, China; ^2^ Clinical Laboratory Department, Yan’an Hospital Affiliated to Kunming Medical University, Kunming, China; ^3^ Department of Emergency, The Traditional Chinese Medicine Hospital of Wenjiang District, Chengdu, China

**Keywords:** CRISPR/Cas12a, loop-mediated isothermal amplification, *Shigella* flexneri, point of care test, visual detection

## Abstract

*Shigella flexneri* is a serious threat to global public health, and a rapid detection method is urgently needed. The CRISPR/Cas (clustered regularly interspaced short palindromic repeats/CRISPR-associated) system is widely used in gene editing, gene therapy, and *in vitro* diagnosis. Here, we combined loop-mediated isothermal amplification (LAMP) and CRISPR/Cas12a to develop a novel diagnostic test (CRISPR/Cas12a-E-LAMP) for the diagnosis of *S. flexneri*. The CRISPR/Cas12a-E-LAMP protocol conducts LAMP reaction for *S. flexneri* templates followed by CRISPR/Cas12a detection of predefined target sequences. LAMP primers and sgRNAs were designed to the highly conserved gene *hypothetical protein* (accession: AE014073, region: 4170556–4171,068) of *S. flexneri*. After the LAMP reaction at 60°C for 20 min, the pre-loaded CRISPR/Cas12a regents were mixed with the LAMP products in one tube at 37°C for 20 min, and the final results can be viewed by naked eyes with a total time of 40 min. The sensitivity of CRISPR/Cas12a-E-LAMP to detect *S. flexneri* was 4 × 10^0^ copies/μl plasmids and without cross-reaction with other six closely related non-*S. flexneri.* Therefore, the CRISPR/Cas12a-E-LAMP assay is a useful method for the reliable and quick diagnosis of *S. flexneri* and may be applied in other pathogen infection detection.

## Introduction

*Shigella* is a kind of Gram-negative bacilli, and it is the most common pathogen of human bacillary dysentery (Jehl et al., 2012), which is a frequently occurring disease in developing countries, and seriously endangers human health, especially the growth and development of children (Wanwu Li et al., 2021). According to the survey of the bacterial distribution of *Shigella*, *Shigella flexneri* is the main pathogen of bacterial diarrhea in developing countries worldwide (Wang et al., 2014). Traditional bacterial culture method combined with biochemical experiments for the identification of *S. flexneri* is time-consuming (Weiss et al., 2019). With the development of molecular biology techniques, the pathogenic mechanism and detection methods of *S. flexneri* have also been further studied and applied (Gentle et al., 2016). The development of sensitive, specific, convenient, and rapid detection kits for *S. flexneri* can provide technical guarantees for livestock and poultry breeding and provide technical support for food safety and human health. Thus, the rapid and accurate detection technology of *S. flexneri* is very important.

CRISPR/Cas (clustered regularly interspaced short palindromic repeats/CRISPR-associated) system is an adaptive immune defense system used by prokaryotes to resist the invasion of foreign genetic materials (Jinek et al., 2012). It is composed of many short and conservative repeats, which transcribed into trans-activating crRNA (tracrRNA); spacers, which transcribed into CRISPR RNA (crRNA); and CRISPR-associated genes, which transcribed into Cas proteins (Karvelis et al., 2013). Cas proteins are recruited by the artificially programmed single-guide RNA (sgRNA) to form a functional ribonucleoprotein complex, and sgRNA is formed by tracrRNA and crRNA (Yang et al., 2020). The sgRNA captures the target by sequence complementation, and Cas proteins cleave the target by the activation of endonuclease activity (cis-cleavage), thus clearing foreign genetic materials and playing an immune-defense role (Wiegand et al., 2020). The sgRNA can be programmed towards a specific DNA or RNA region of interest through hybridization to a complementary sequence, which in some systems is restricted to the proximity of a protospacer adjacent motif (PAM) or protospacer flanking sequence (Bolotin et al., 2005). In theory, CRISPR/Cas system can recognize any sequences by controlling the sequence of sgRNA, which is the basis for its wide application in gene editing and molecular diagnosis.

At present, numerous CRISPR/Cas endonucleases have been identified, which could be divided into two classes, class 1 and 2. Class 1 includes three types (types I, III, and IV) and 22 subtypes of CRISPR/Cas systems, and class 2 includes three types (types II, V, and VI) and 26 subtypes (Jolany Vangah et al., 2020). Diagnostic CRISPR/Cas systems are often derived from types V (Cas12 and Cas14) and VI (Cas13) in class 1. Once the target is cleavaged by the Cas proteins, the trans-cleavage or collateral cleavage is activated, which results in the cleavage of any nearby single-stranded DNA (ssDNA) probes (fluorescent and quenching markers) (Kaminski et al., 2021). Cas12 can cleave double strands of DNA under RNA guidance and collaterally cleaves ssDNA probe, and Cas13 can cleave RNA targets under RNA guidance and collaterally cleaves ssRNA probe, while Cas14 can cleave ssDNA and dsDNA and collaterally cleaves ssDNA probe (Karvelis et al., 2020; Kaminski et al., 2021; Takeda et al., 2021). Although CRISPR/Cas has the potential for accurate diagnosis, a sufficient amount of target is still needed for the IVD method to ensure detection sensitivity. Various nucleic acid amplification techniques have been used to produce enough and specific targets, including polymerase chain reaction (PCR) (S Wang et al., 2021), loop-mediated isothermal amplification (LAMP) (Crone et al., 2020), recombinase polymerase amplification (RPA) (Xianfeng Wang et al., 2021), rolling circle amplification (RCA) (Ruixuan Wang et al., 2020), crossing priming amplification (CPA) (Wang et al., 2019), strand displacement amplification (SDA) (Dong-Xia Wang et al., 2020), and nucleic acid sequence-based amplification (NASBA) (Ju et al., 2021).

Based on the cis-cleavage and/or trans-cleavage of Cas proteins and the nucleic acid amplification technique, many CRISPR/Cas diagnostic methods have been developed. Pardee et al. (2016) successfully developed the NASBA-CRISPR/Cas9 method to distinguish American and African ZIKV strains, with single-base resolution. Huang et al. (2018) employed a CRISPR/Cas9-triggered isothermal exponential amplification reaction (CAS-EXPAR) strategy, to detect DNA targets with attomolar sensitivity and single-base specificity. Gootenberg et al. (2017) established a specific high-sensitivity enzymatic reporter unlocking (SHERLOCK) platform (RPA-CRISPR/Cas13a method) to detect the specific strains of zika and dengue viruses, to distinguish pathogenic bacteria and mutations in cell-free tumor DNA. Based on the RPA-CRISPR/Cas12a method, DETECTR (DNA Endonuclease Targeted CRISPR Trans-Reporter) and HOLMES (a one-HOur Low-cost Multipurpose highly Efficient System) were also established to detect genotypes of HPV strains in patient samples (Chen et al., 2018; Li et al., 2018). Rui Wang et al. (2021) developed the opvCRISPR (one-pot visual RT-LAMP-CRISPR) platform for the detection of SARS-CoV-2 within 45 min at nearly a single-molecule level. Harrington et al. (2018) integrated Cas14a into the DETECTR platform to generate a new ssDNA detection system termed Cas14a-DETECTR, which could be used in the detection of single-nucleotide polymorphisms without PAM constraint. However, due to the different operating temperatures of nucleic acid amplification techniques and CRISPR/Cas and the complex components of the system, CRISPR/Cas-based diagnostic methods were difficult to finish in one simple system. Typically, it requires separate pre-amplification of nucleic acid and multiple manual operations, which undoubtedly complicates the testing procedures and potentially increases the risk of carry-over contaminations due to the process of amplification product transferring.

**FIGURE 1:**
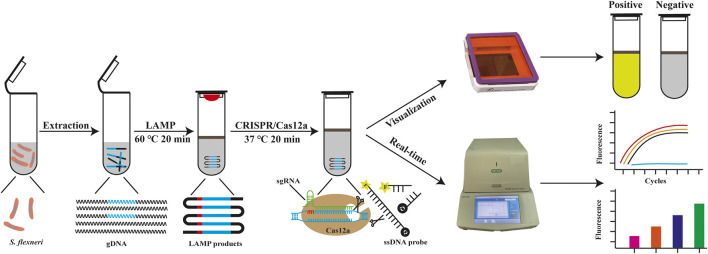
The outline of CRISPR/Cas12a-E-LAMP detection system. In the CRISPR/Cas12a-E-LAMP method, the gDNA of *S. flexneri* was extracted and then used in the LAMP reaction (10 μl) at 60°C for 20 min and covered by 20-μl mineral oil. The Cas12a reaction reagents (20 μl) was pre-placed in the lid and mixed with LAMP products at 37°C for 20 min. Once the Cas12a find the PAM site and the sgRNA complementary pairing with the target DNA, Cas12a endonuclease is activated. Then, Cas12a endonuclease cis-cleavage the LAMP products and split the ssDNA-FQ reporter indiscriminately by trans-cleavage, generating the fluorescence signal visible to the naked eye under LED blue light.

In this study, we developed a one-tube CRISPR/Cas12a-enhanced LAMP (CRISPR/Cas12a-E-LAMP) method for the visual detection of *S. flexneri*, which simplified the operations and avoided aerosol contamination. In this CRISPR/Cas12a-E-LAMP, the LAMP reagents are added at the bottom of the tube, and the CRISPR/Cas12a reaction reagents are added on the lid. *S. flexneri* DNA templates are amplified by LAMP reaction, followed by upside-down mixing with the CRISPR/Cas12a reagents for cleavage. Once the Cas12a nuclease is activated by recognizing DNA target, it splits the ssDNA-FQ indiscriminately, generating the fluorescence signal visible to the naked eye under LED blue light (Figure 1). In addition, the CRISPR/Cas12a-E-LAMP reaction could be monitored by the CFX96 Touch Real-Time PCR Detection System. Therefore, CRISPR/Cas12a-E-LAMP has a potential application in the resource-constrained environment for the rapid detection of pathogens of interest.

## Materials and Methods

### Materials

Primers, ssDNA-FQ probe, sgRNAs, and agarose were obtained from Tsingke Biotechnology Co., Ltd. (Beijing, China); Bst 3.0 DNA polymerase and EnGen^®^Lba Cas12a (Cpf1, from *Lachnospiraceae bacterium ND 2006*) were obtained from New England Biolabs Inc. (Massachusetts, United States); RNase inhibitor, TGreen Transilluminator (LED blue light), TIANamp Bacteria DNA Kit, and TIANprep Mini Plasmid Kit were obtained from TIANGEN Biotech (Beijing) Co., Ltd. (Beijing, China); UVP UVsolo Touch was purchased from Analytik Jena AG (Jena, Germany); dry bath incubator was purchased from Hangzhou Miu Instruments Co., Ltd. (Hangzhou, China), EasyTaq^®^ PCR Super Mix, pEASY^®^-T1 Cloning Kit, and qPCR premix were obtained from TransGen Biotech Co., Ltd. (Beijing, China); the CFX96 Touch Real-Time PCR Detection System was purchased from Bio-Rad Laboratories, Inc. (California, United States); NanoDrop UV-Vis spectrophotometer, Applied Biosystems VeritiPro PCR, Luria-Bertani (LB) liquid medium, mineral oil, and routine biochemical reagents were purchased from Thermo Fisher Scientific Inc. (Shanghai, China). Twenty *S. flexneri* clinical isolates and six non-*S. flexneri* clinical isolates including *Enterococcus faecalis*, *Salmonella enteritidis*, *Klebsiella pneumoniae*, *Proteus mirabilis*, *Escherichia coli*, and *Staphylococcus aureus* used in this study were identified by the fully automatic VITEK^®^2 Compact system (BioMérieux, Warszawa, Poland) and kindly provided by the Yan’an Hospital Affiliated to Kunming Medical University (Yunnan, China). All bacterial strains were cultured at 37°C and 180 rpm overnight in the LB liquid medium. Bacterial genome DNA (gDNA) was extracted using TIANamp Bacteria DNA kit according to the manufacturer’s instructions and stored at −20°C until use.

## Primers, sgRNAs, ssDNA Probe Designing, and Plasmid Construction

The highly conserved gene *hypothetical protein* of *S. flexneri* used in this study was screened as described in our previous study (Shi et al., 2021). LAMP primers were designed by the Primer Explorer V5 software (http://primerexplorer.jp/lampv5/index.html) and synthesized by Tsingke Biotechnology Co., Ltd. The sgRNAs were designed in CHOPCHOP (http://chopchop.cbu.uib.no/) based on the conserved gene. The FAM/BHQ-labeled ssDNA probe was determined based on the Cas12a enzyme used in this study. The sequence information of all primers, sgRNAs, and ssDNA probes used are listed in Supplementary Table S1. To obtain the standard template, plasmids containing the amplification target of the *hypothetical protein* gene were constructed, which is described in the Supplementary Material.

### PCR/qPCR/LAMP Amplification Reaction

The PCR reaction system contains 12.5 μl EasyTaq^®^PCR Super Mix, 0.4 μM of the primers, and 1 μg gDNA template or 1 μl of the gradient dilution recombinant plasmid, adding nuclease-free water up to 25 μl. Then, PCR reaction was performed in an Applied Biosystems VeritiPro PCR with the following amplification steps: pre denaturation at 94°C for 5 min, followed by 30 cycles of denaturation at 94°C for 30 s, annealing at 58°C for 30 s, extension at 72°C for 30 s, and a final extension at 72°C for 7 min. For qPCR amplification, 20 μl of the qPCR reaction mixture, composed of 0.2 μM of the primers, 10 μl of premix, 1 μg gDNA template or 1 μl of the gradient dilution recombinant plasmid, and ddH_2_O was added up to 20 μl. The reaction mixture was incubated in a CFX96 Touch Real-Time PCR Detection System using a three-step qPCR protocol: pre denaturation at 95°C for 30 s, followed by 40 cycles of denaturation at 95°C for 5 s, annealing at 58°C for 15 s, and extension at 72°C for 10 s. The optimized reaction system of LAMP contained 1 μl of Isothermal Amplification Buffer II, 6 mM Mg^2+^, 320 U/ml of Bst 3.0 DNA polymerase, 1.2 mM deoxyribonucleotide (dNTPs), 0.2 μM of the outer primer (F3/B3), 1.6 μM of the inner primer (FIP/BIP), 1 μg gDNA template or 1 μl of the gradient dilution recombinant plasmid, and nuclease-free water up to 10 μl. This mixture was incubated at 60°C in a dry bath incubator for 20 min. To reduce heat transfer and prevent contamination, 20 μl of mineral oil was added to cover the 10-μl LAMP reaction mixture. Both the PCR and LAMP products were verified using gel electrophoresis on a 3% agarose gel at 120 V for 30 min and visualized under a UV transilluminator (UVP UVsolo Touch).

### Formation of CRISPR/Cas12a-E-LAMP Detection System

CRISPR/Cas12a-E-LAMP detection system was composed of LAMP reaction and CRISPR/Cas12a reaction. The 10-μl LAMP reaction mixture was added to the bottom of the tube and covered by 20-μl mineral oil to avoid the heat transfer and contamination. Then, 20 μl of CRISPR/Cas12a-optimized reaction mixture was added into the inside lid of the tube, which contains 1*NEBuffer 2.1 Reaction Buffer, 200 nM EnGen^®^Lba Cas12a (Cpf1), 0.83 μM gRNA, 1.17 μM ssDNA probes, 4 U/μl RNA inhibitor, and nuclease-free water. After LAMP reaction at 60°C in a dry bath incubator for 20 min, 20 μl of CRISPR/Cas12a-optimized reaction mixture was mixed with the LAMP amplification solution by hand shaking. The tube was put in the dry bath incubator at 37°C for 20 min, and the endpoint fluorescence was observed under the LED blue light (TGreen Transilluminator). To optimize and better monitor the process of CRISPR/Cas12a reaction, CFX96 Touch Real-Time PCR Detection System was used for the real-time monitoring (37°C for 1 min, 30 cycles).

### Specificity and Sensitivity Evaluation of CRISPR/Cas12a-E-LAMP System

To verify the specificity of our CRISPR/Cas12a-E-LAMP system, *S. flexneri* was used as a positive control strain, and six non-*S. flexneri* (*E. faecalis*, *S. enteritidis*, *K. pneumoniae*, *P. mirabilis*, *E. coli*, and *S. aureus*) were used as negative controls, and ddH_2_O was used as a no-template control and compared with the gold standard method qPCR. To verify the sensitivity of CRISPR/Cas12a-E-LAMP method, serial 10-fold dilutions of *S. flexneri* recombinant plasmids (4 × 10^5−0^ copies/μl) were used as the templates, compared with PCR, qPCR, and LAMP, respectively.

### Clinical Evaluation of CRISPR/Cas12a-E-LAMP

Twenty *S. flexneri* clinical isolates were identified by the fully automatic VITEK^®^2 Compact system and donated from the Yan’an Hospital Affiliated to Kunming Medical University, which were used in the clinical evaluation of CRISPR/Cas12a-E-LAMP. gDNA of the bacterial strains were extracted and employed for validating this protocol and compared with the gold standard method qPCR.

## Results

### The Working Principle of CRISPR/Cas12a-E-LAMP Detection System

The working principle of CRISPR/Cas12a-E-LAMP is shown in Figure 1. The target sequence of LAMP amplification was screened and obtained as previously described, which was a highly conserved sequence in *S. flexneri* and could be used as a diagnostic marker for the identification of *S. flexneri.* LAMP primers were designed according to the conserved sequence *hypothetical protein* (accession: AE014073, region: 4170556–4171,068), and sgRNAs were designed based on the amplification products of LAMP; the used primer and sgRNA binding sites are shown in Supplementary Figure S1. The gDNA of *S. flexneri* was used for LAMP amplification (65°C, 20 min), and the LAMP amplification was covered by the mineral oil, which was used to avoid the generation of aerosol and reduce the spread of heat. After LAMP reaction, CRISPR/Cas12a cleavage system was mixed with the LAMP products in a tube, Cas12a cis-cleavage the products of LAMP under the guide of sgRNA (37°C, 20 min), and trans-cleavage the ssDNA probe to generate the bright fluorescence. The results could be observed by the naked eyes under the LED blue light or be monitored by the CFX96 Touch Real-Time PCR detection system.

### Construction of LAMP Reaction

Enough amount of high-quality LAMP products is a prerequisite for the CRISPR/Cas12a-E-LAMP detection system. Therefore, the LAMP reaction was built and optimized from multi-aspects including temperature, Mg^2+^, dNTPs, time, and reaction volume, and 4 × 10^3^ copies/μl recombinant plasmid was used as the template (Supplementary Figure S2). The reaction temperature is vital to the LAMP reaction, which was optimized from 60°C to 65°C; Supplementary Figure S2A shows that better LAMP products were produced at 60°C. Mg^2+^ is the key factor to ensuring Bst enzyme activity, which was optimized from 2 to 12 mM, and Supplementary Figure S2B shows that better LAMP products were produced at 6 mM. dNTPs were the substrate for nucleic acid synthesis. Excessive dNTPs will chelate with Mg^2+^ and affect the activity of Bst enzyme; the dNTP concentrations were optimized from 0.8 to 2.0 mM. Supplementary Figure S2C shows that better LAMP products were generated at 1.2 mM dNTPs. For CRISPR/Cas12a-E-LAMP detection system, shortening LAMP amplification time is beneficial to shorten the overall time of CRISPR/Cas12a-E-LAMP while ensuring the production of enough LAMP products. As shown in Supplementary Figure S2D, LAMP amplification time was optimized from 10 to 60 min, and LMAP products could be detected by the agarose gel electrophoresis after 20 min, which was used in the following study. In the case of ensuring the amplification efficiency of LAMP, shortening the volume of LAMP saves costs and has a better clinical application potential. As shown in Supplementary Figure S2E, LAMP amplification volume was optimized from 5 to 30 μl, and 10 μl is enough to guarantee the LAMP amplification efficiency.

### Establishment of CRISPR/Cas12a-E-LAMP Detection Method

**FIGURE 2:**
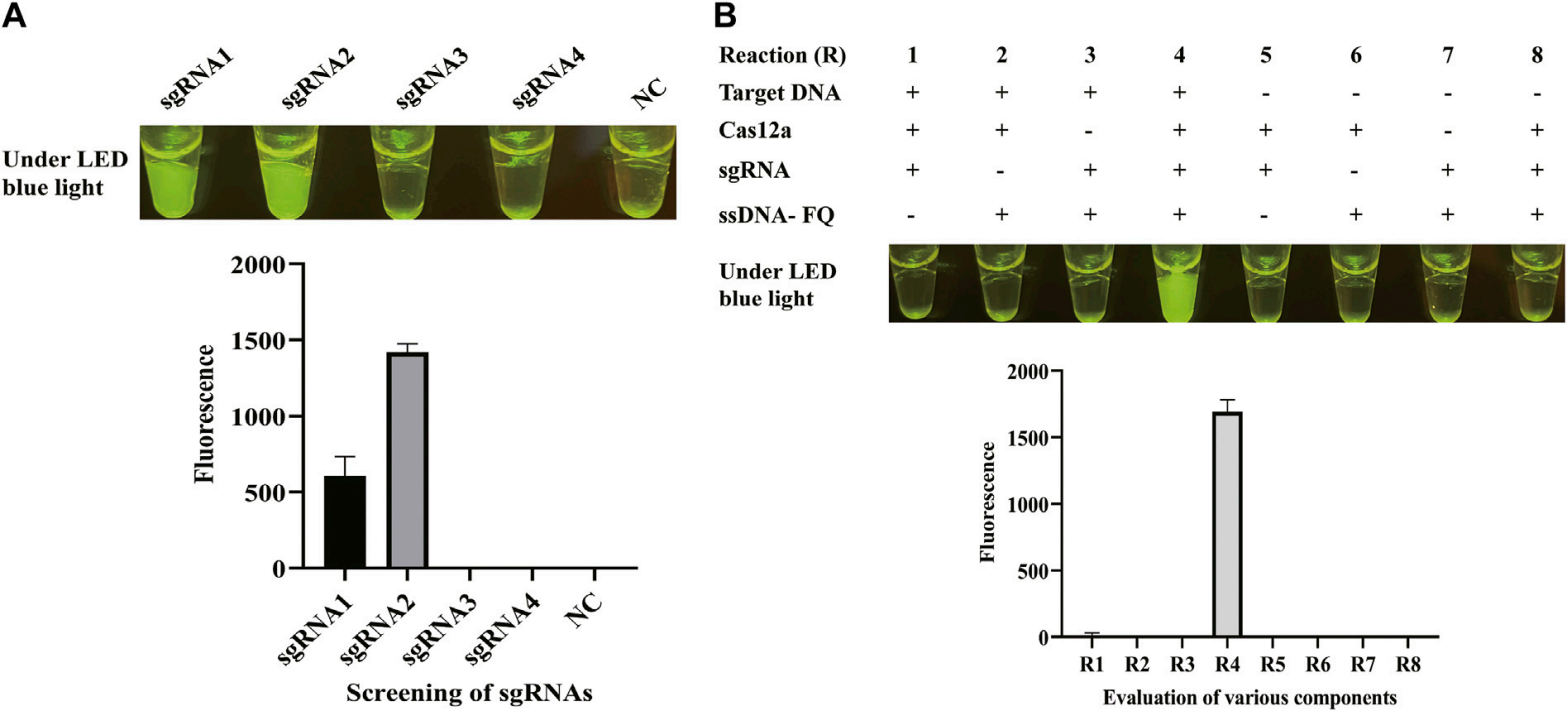
sgRNA screening and evaluation of various components. **(A)** Four sgRNAs were designed based on the target sequence and used for the screening of sgRNAs in the CFX96 Touch Real-Time PCR Detection System. The results were shown by endpoint fluorescence photography and endpoint fluorescence intensity; NC, negative control. **(B)** Various components were evaluated through endpoint fluorescence photography after 30-min incubation and real-time fluorescence detection. The LAMP products, Cas12a, sgRNA, and ssDNA-FQ reporter were tested. The results were shown by endpoint fluorescence photography and endpoint fluorescence intensity.

**FIGURE 3:**
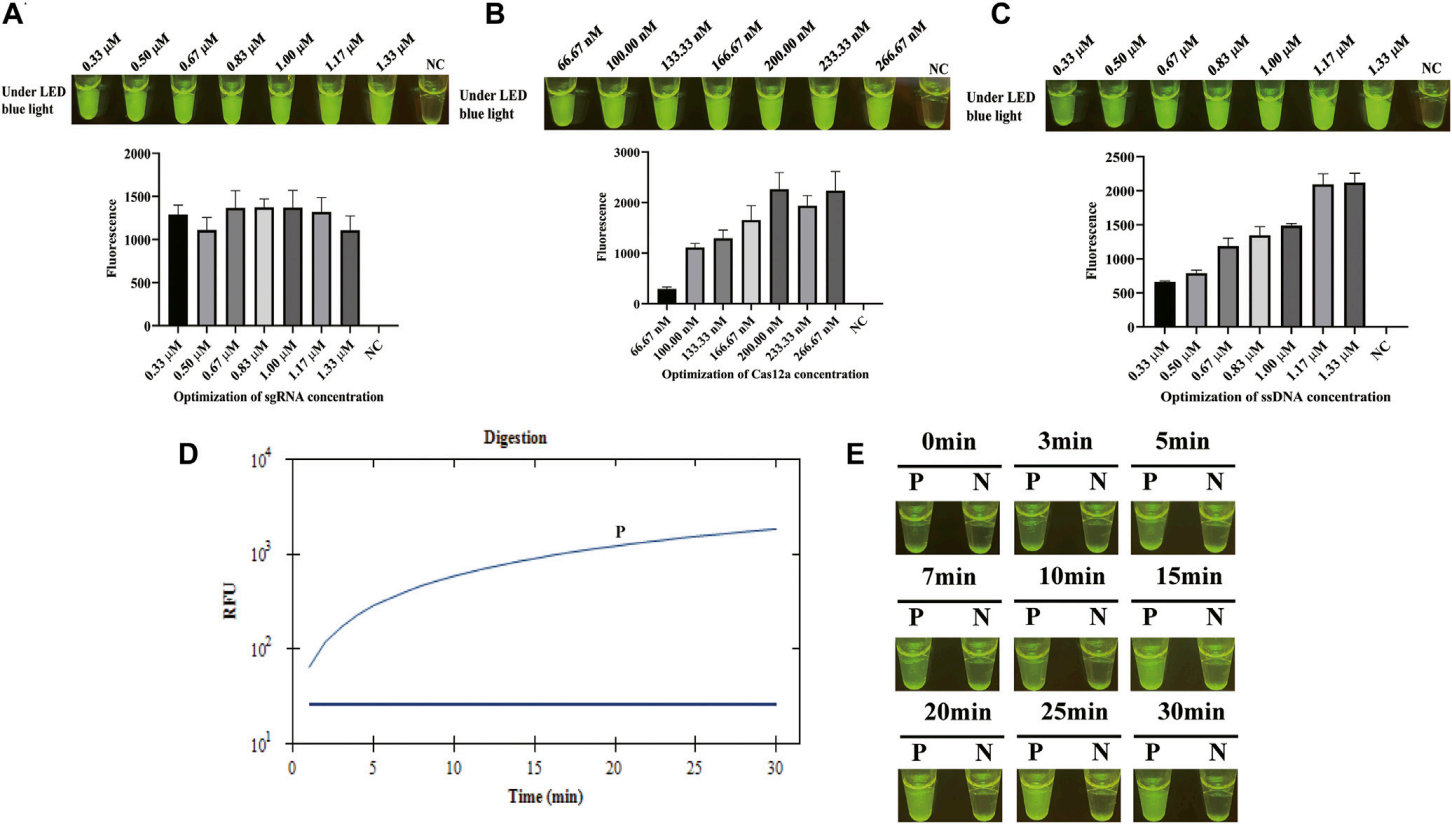
Optimization of CRISPR/Cas12a-E-LAMP method. *S. flexneri*-recombined plasmids (4*10^3^ copies/μl) were used in the optimization of CRISPR/Cas12a-E-LAMP method by endpoint fluorescence photography and endpoint fluorescence intensity. **(A)** Optimization of the concentration of sgRNA. **(B)** Optimization of the concentration of Cas12a. **(C)** Optimization of the concentration of ssDNA-FQ probe. **(D,E)** The time optimization of CRISPR/Cas12a-E-LAMP.

Once the LAMP amplification was finished, CRISPR/Cas12a mixture was mixed with LAMP products for cleavage. To obtain a better cleavage activity of CRISPR/Cas12a, it was optimized from sgRNA, Cas12a, and ssDNA probe. sgRNA plays a critical role of guidance in CRISPR/Cas12a cleavage system. sgRNA was composed of a sequence for the recognition of the target sequence and a sequence that forms a complex with Cas12a by stem ring structure (Supplementary Figure S1). Four sgRNAs were designed based on the target sequence and used for the screening of sgRNAs in CFX96 Touch Real-Time PCR Detection System. Figure 2A shows that sgRNA2 exhibited significant and stable efficacy in guiding cleavage and has a significantly higher fluorescence. To clarify the reagent composition factors that affect fluorescence generation, the reagent composition of CRISPR/Cas12a-E-LAMP reaction system was optimized. As Figure 2B shows, only when the target DNA, Cas12a, sgRNA, and ssDNA were in the reaction system will the strong fluorescence be produced. Then, the concentration of sgRNA was optimized from 0.33 to 1.33 μM, and the highest fluorescence intensity was obtained at 0.83 μM (Figure 3A). In addition, Cas12a plays the role of scissors under the guidance of sgRNA, which can cis-cleave the target-specific and then collaterally cleaves on ssDNA probe (trans-cleavage). The concentration of Cas12a and ssDNA probes were very important; thus, the concentration of Cas12a and ssDNA probes after the screening of sgRNA was optimized. Figure 3B, C shows that the highest fluorescence intensity was generated at 200 nM Cas12a and 1.17 μM ssDNA probe. Similar to the LAMP reaction optimization, shortening CRISPR/Cas12 cleavage time is beneficial to shorten the overall time of CRISPR/Cas12a-E-LAMP while ensuring the production of distinguishable fluorescence intensity. Thus, the cleavage time was optimized from 0 to 30 min, and 20 min was chosen for the cleavage time as determined by fluorescence intensity (Figure 3D, E).

### Specificity and Sensitivity Evaluation of the CRISPR/Cas12a-E-LAMP System

**FIGURE 4:**
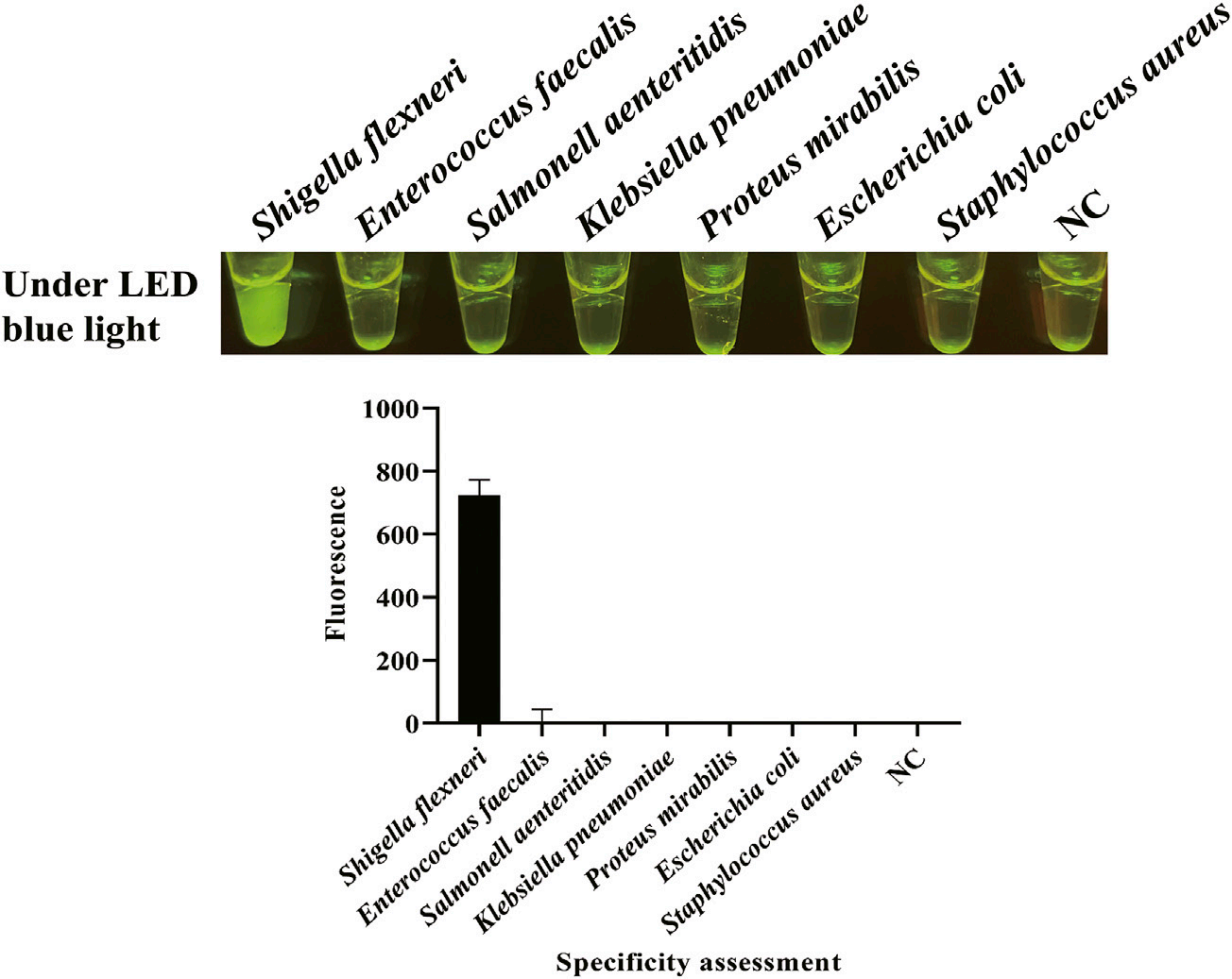
Specificity evaluation of CRISPR/Cas12a-E-LAMP. gDNA of *S. flexneri* and non-*S. flexneri* (*E. faecalis*, *S. enteritidis*, *K. pneumoniae*, *P. mirabilis*, *E. coli*, and *S. aureus*) were used as the template for the specificity evaluation of CRISPR/Cas12a-E-LAMP by endpoint fluorescence photography and endpoint fluorescence intensity.

**FIGURE 5:**
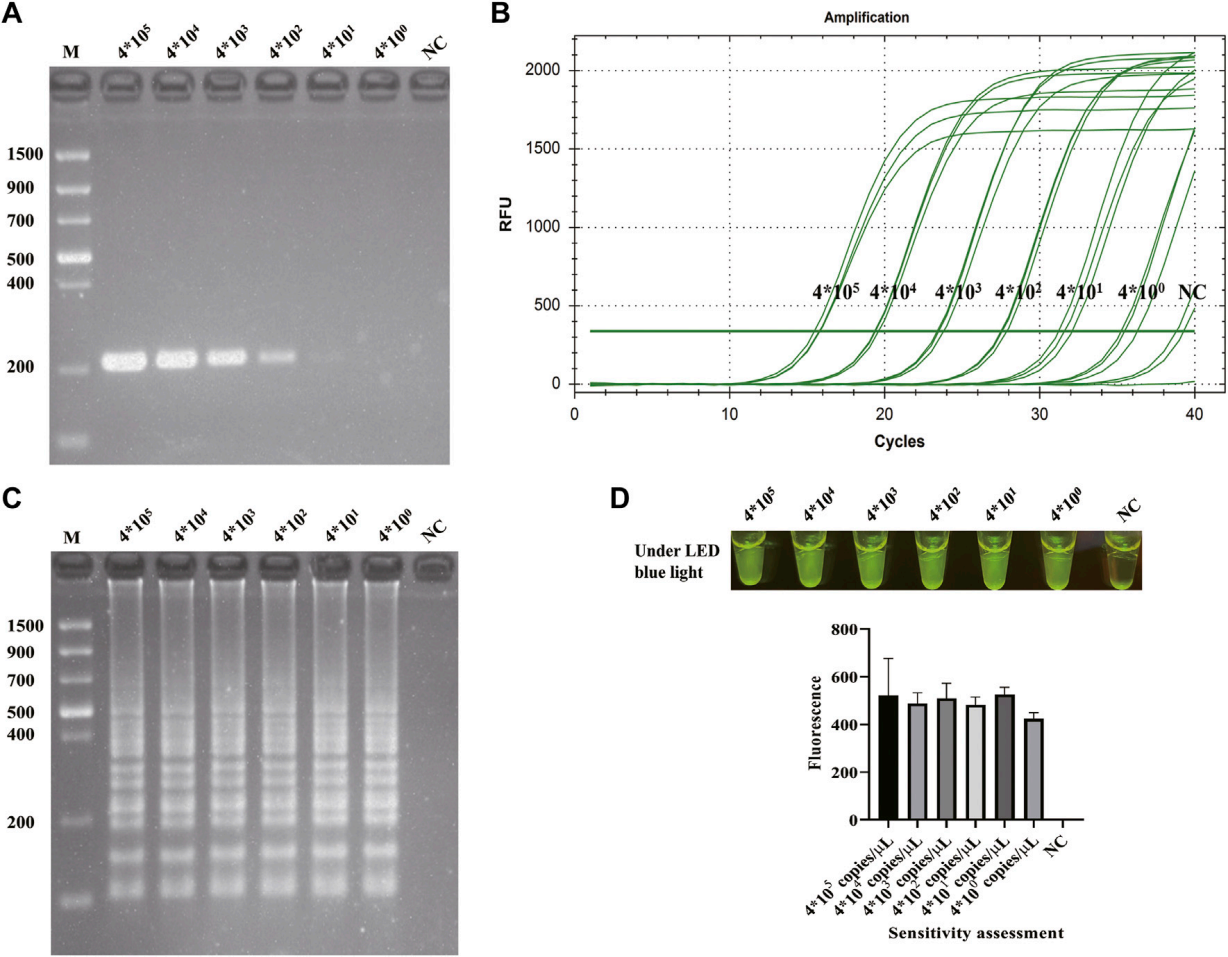
Sensitivity evaluation of CRISPR/Cas12a-E-LAMP. The sensitivity of CRISPR/Cas12a-E-LAMP was compared with PCR, qPCR, and LAMP. The serial 10-fold dilutions of *S. flexneri*-recombined plasmids (4*10^5−0^ copies/μl) were used for the sensitivity evaluation. The sensitivity evaluation results of PCR and LAMP were shown by agarose gel electrophoresis. The sensitivity evaluation results of qPCR and CRISPR/Cas12a-E-LAMP were shown by the real-time amplification curve, endpoint fluorescence photography, and endpoint fluorescence intensity. **(A)** The sensitivity evaluation of PCR. **(B)** The sensitivity evaluation of qPCR. **(C)** The sensitivity evaluation of LAMP. **(D)** The sensitivity evaluation of CRISPR/Cas12a-E-LAMP. NC, negative control.

After the optimization of LAMP reaction and CRISPR/Cas12a cleavage reaction and the establishment of CRISPR/Cas12a-E-LAMP method, the specificity and sensitivity of this method were evaluated. Because the specificity of the *hypothetical protein*-encoding gene was confirmed in our previous study by a WarmStart colorimetric LAMP method (Shi et al., 2021), we focused more on the sensitivity evaluation. gDNA of *S. flexneri* and non-*S. flexneri* (*E. faecalis*, *S. enteritidis*, *K. pneumoniae*, *P. mirabilis*, *E. coli*, and *S. aureus*) were used as the template for the specificity evaluation of CRISPR/Cas12a-E-LAMP system. Figure 4 shows that the *S. flexneri* can be easily identified by this CRISPR/Cas12a-E-LAMP method without cross reaction with the non-*S. flexneri.* Supplementary Table S2 shows the specificity detection results of qPCR, and all the non-*S. flexneri* were fluorescence negative. To verify the sensitivity of the CRISPR/Cas12a-E-LAMP method, serial 10-fold dilutions of *S. flexneri* recombinant plasmids (4*10^5−0^copies/μl) were used as the templates, and they were compared with PCR, qPCR, and LAMP. As shown in Figure 5, the limit of detection of PCR (Figure 5A) was 4*10^1^ copies/μl, while the limit of detection of qPCR (Figure 5B), LAMP (Figure 5C), and CRISPR/Cas12a-E-LAMP (Figure 5D) was 4*10^0^ copies/μl.

### Performance Evaluation of CRISPR/Cas12a-E-LAMP Using *S. flexneri* Clinical Isolates

**FIGURE 6:**
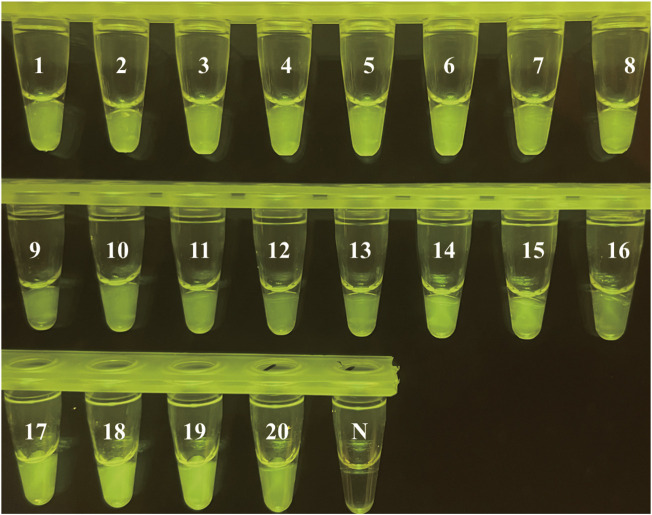
*S. flexneri* isolate evaluation using CRISPR/Cas12a-E-LAMP. Twenty *S. flexneri* strains were used in the clinical evaluation of CRISPR/Cas12a-E-LAMP.

To explore the feasibility of this method in practical use, the gDNA of 20 *S. flexneri* clinical isolates were extracted and used in the LAMP reaction at 60°C for 20 min, then mixed with the pre-loaded CRISPR/Cas12a regents in the tube lid at 37°C for 20 min. Also, the extracted gDNA of 20 *S. flexneri* clinical isolates were detected by the gold standard of qPCR. Figure 6 shows that all the *S. flexneri* clinical isolates were identified by our CRISPR/Cas12a-E-LAMP, as the generation of fluorescence means the positive detection. Also, all the *S. flexneri* clinical isolates were positively detected by the qPCR (Supplementary Table S2).

## Discussion

It has been almost 40 years since the discovery of CRISPR/Cas, which is a type of adaptive immune system found in bacteria and archaea that naturally protects against the invading nucleic acids (Ishino et al., 1987). At present, CRISPR/Cas plays an important role not only in immunity but also in gene editing, gene therapy, and especially in IVD (Kaminski et al., 2021). In IVD, a large number of culture-, biochemical-, serological-, and molecular-based (nucleic acid) diagnostic methods were established for the detection of pathogens, and molecular diagnosis has gradually replaced the traditional biochemical and serological detection and occupies a higher market share in the field of IVD (Min Li et al., 2021). Nucleic acid amplification in molecular diagnostics can be divided into constant-temperature amplification (e.g., LAMP and RPA) and variable-temperature amplification (e.g., PCR and qPCR) according to the temperature. Variable-temperature amplification requires precise temperature-control instruments to provide the changing temperature to perform nucleic acid amplification reactions. Constant-temperature amplification allows nucleic acid amplification at a constant temperature, which is non-instrument dependent. CRISPR/Cas, especially Cas12, Cas13, and Cas14, were widely used in molecular diagnosis combined with nucleic acid amplification (Min Li et al., 2021), and as a novel molecular tool, it has great potential in IVD applications.

Although qPCR is the gold standard and widely used for pathogen detection with high sensitivity and reliability, it is not very suitable for POCT due to sophisticated equipment-dependent and long reaction time (Rodriguez-Manzano et al., 2021). The proposed CRISPR/Cas12a-E-LAMP method integrates LAMP and CRISPR/Cas12a in one tube with a similar limit of detection to qPCR. The characteristic of LAMP is that the four primers are designed according to the six regions of a target gene or sequence, which could greatly ensure the specificity of amplification. Strand displacement Bst DNA polymerase is involved in the continuous self-cycle synthesis of strand displacement DNA by four primers to achieve rapid (15–60 min) and sensitive amplification (Notomi et al., 2000; Zhu et al., 2020). Thus, the proposed CRISPR/Cas12a-E-LAMP method has great potential to enable POCT outside of the clinical diagnostic laboratory, such as airports, local emergency departments and clinics, and other locations.

*S. flexneri* was chosen as the subject for CRISPR/Cas12a-E-LAMP method. *S. flexneri* is a kind of Gram-negative short *bacillus*, which is the most common pathogen of bacillary dysentery in humans, and it is mainly prevalent in developing countries (Chang et al., 2020). Bacillary dysentery is the most common intestinal infectious disease, with the most patients appearing in summer and fall (Liu et al., 2016). The main source of infection is contaminated food, drinking water, and other oral infections (Kuti et al., 2020). Humans are susceptible to *S. flexneri*, and less than 10 bacteria can cause human infection, so *S. flexneri* is highly contagious and harmful (Cao et al., 2021). The traditional detection method for *S. flexneri* is mainly based on the physiological and biochemical characteristics of the bacteria, which often takes a long time, and it is not conducive to timely finding pathogens and diagnosing causes (Chang et al., 2014; Opota et al., 2015; Magalhaes et al., 2021). Accordingly, a fast, accurate, and relatively inexpensive detection method for *S. flexneri* is of great demand, especially in hospitals and laboratories with poor medical facilities and in situations where an urgent diagnosis is needed.

In this study, a CRISPR/Cas12a-E-LAMP method for the detection of *S. flexneri* was developed*.* The DNA of *S. flexneri* was used as a template in the LAMP reaction first to generate enough target products, then mixed with CRISPR/Cas12a system that was pre-loaded in the lid of the tube for cleavage. The fluorescence was observed by naked eyes under LED, and blue light means the positive detection of *S. flexneri*. The sensitivity of CRISPR/Cas12a-E-LAMP was 4*10^0^ copies/μl recombinant plasmids, without cross-reaction with other regularly non-*S. flexneri*. The total time of CRISPR/Cas12a-E-LAMP detection was 40 min, which comprises 20 min for LAMP reaction and 20 min for CRISPR/Cas12 cleavage. We demonstrated that the cleavage of fluorescence-quenched reporters by CRISPR/Cas12a can make a second-round signal amplification, which decreases the detection time and makes this method more distinguishable with the naked eye than colorimetric methods or agarose gel electrophoresis. Also, the specificity of the CRISPR/Cas12a-E-LAMP was enhanced by the designed specific sgRNA in the CRISPR/Cas12a system and the designed specific LAMP primers in LAMP reaction, which could guarantee the accurate detection of *S. flexneri* and has unique methodological advantages than the traditional detection methods. Besides, this CRISPR/Cas12a-E-LAMP method has the advantages of non-instrument dependence, and all the CRISPR/Cas12a-E-LAMP operations could be finished in a small place by one simple constant temperature bath, even a water bath. Thus, our CRISPR/Cas12a-E-LAMP method shows that the CRISPR/Cas12a has great potential in the development of next-generation biosensors for nucleic acid detection, owing to the trans-cleavage capabilities of the Cas effector proteins.

## Conclusion

In this study, we report the development of a CRISPR/Cas12a-enhanced LAMP detection technology for rapid detection of *S. flexneri*, named CRISPR/Cas12a-E-LAMP assay. The result can be visualized by the naked eyes under the LED blue light, and the whole test was completed within 40 min. The data of analytical sensitivity, specificity, and clinical evaluation initially validated that our protocol is able to accurately differentiate *S. flexneri* from non-*S. flexneri* and reliably diagnose *S. flexneri* infection in clinical isolates. Thus, these traits of this CRISPR/Cas12a-E-LAMP assay address the need for quick diagnosis of the *S. flexneri* in a variety of settings including field, clinical, and resource-limited environments. Also, the CRISPR/Cas12a-E-LAMP assay indicates that CRISPR/Cas12a combined with LAMP has great potential in the development of next-generation biosensors for nucleic acid detection.

## Data Availability

The original contributions presented in the study are included in the article/Supplementary Material, further inquiries can be directed to the corresponding authors.
