# Supplementary material for: CRISPR/Cas12a-Enhanced Loop-Mediated Isothermal Amplification for the Visual Detection of Shigella flexneri
Source: Front Bioeng Biotechnol. 2022 Feb 21;10:845688. doi: 10.3389/fbioe.2022.845688 (PMC8899461; doi:10.3389/fbioe.2022.845688)
Supplement: Supplementary file 1 [file DataSheet1.docx]

Supplementary Material

**Plasmid construction**

To obtain the standard template, plasmids containing the amplification target of *hypothetical protein* gene were constructed by using the pEASY^®^-T1 Cloning Kit. Then the recombinant plasmids were purified with TIANprep Mini Plasmid Kit and verified via sequencing by Tsingke Biotechnology Co., Ltd (Chengdu, China). The copies of recombinant plasmids were calculated by the deduced polynomial model described as Eq:

$$C=\frac{X\times10^{-9}}{\left( 3928+Y \right)\times660}\times N_{A}$$

Where C is the copy of the recombinant plasmid (copies/μL), X and Y represented the concentration of the recombinant plasmids and the number of base pairs in the target fragment, respectively. N_A_ is Avogadro's constant, and 3928 is the number of base pairs in vector, and 660 is the average molecular weight of one base pair.

**Figure S1 The used primer and sgRNA binding sites.**

**
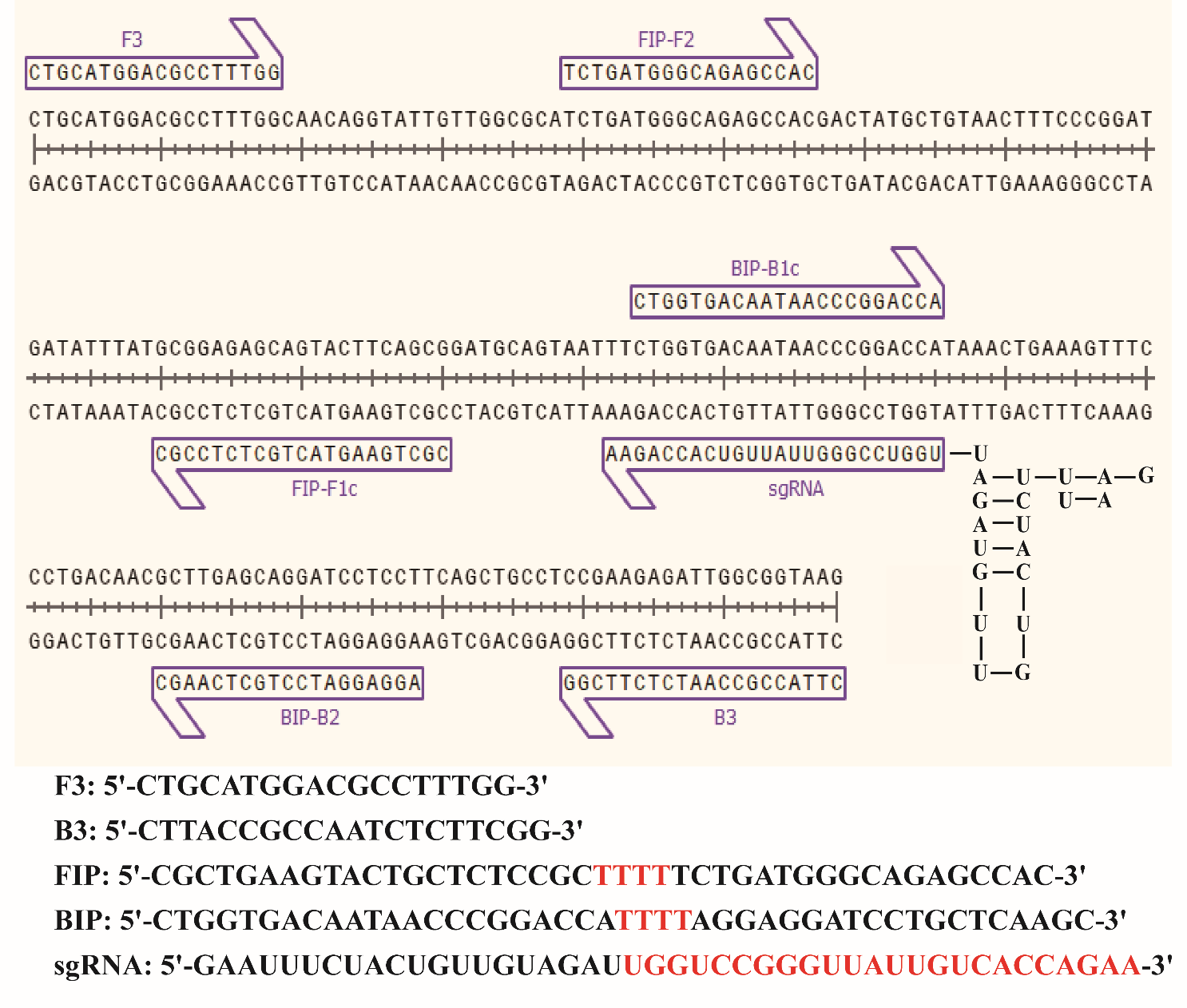
**

**Figure S2** **Construction and** **optimization of LAMP reaction.** Temperature, Mg^2+^, dNTPs, time, and volume evaluation optimized in the LAMP reaction.


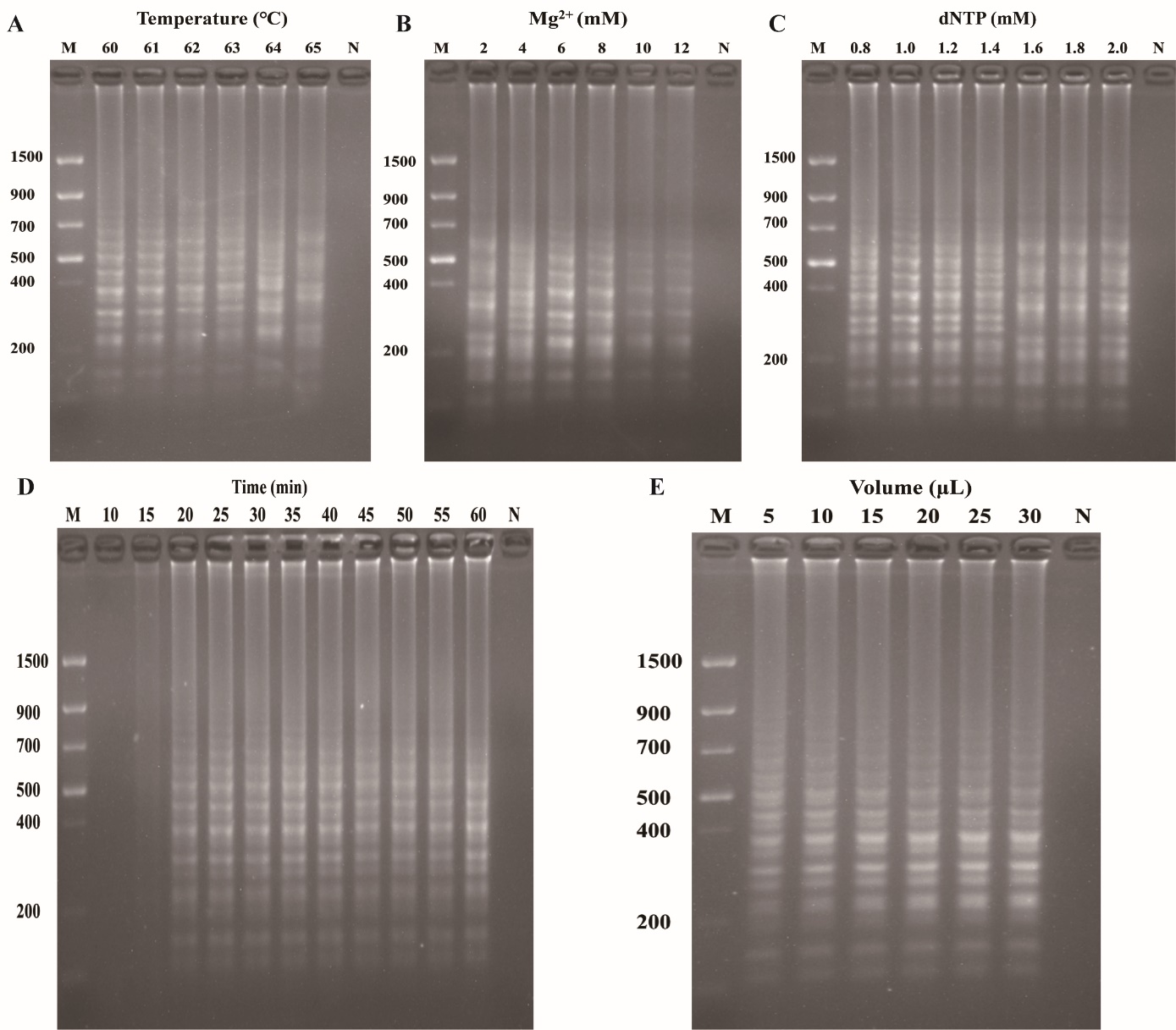


Table S2 Bacterial strains used and results for qPCR and CRISPR/Cas12a-E-LAMP assay for

detection of *S. flexneri*

|  |  | **No. of positive for** | |
| --- | --- | --- | --- |
| **Bacterial species** | **No. tested** | **qPCR** | **CRISPR/Cas12a-E-LAMP** |
| *S. flexneri* | 20 | 20 | 20 |
| **non-*S. flexneri*** |  |  |  |
| *E. faecalis* | 1 | 0 | 0 |
| *S. enteritidis* | 1 | 0 | 0 |
| *K. pneumoniae* | 1 | 0 | 0 |
| *P. mirabilis* | 1 | 0 | 0 |
| *E. coli* | 1 | 0 | 0 |
| *S. aureus* | 1 | 0 | 0 |
